# Impact of Mechanical Complications on Success of Dental Implant Treatments: A Case–Control Study

**DOI:** 10.1055/s-0041-1732802

**Published:** 2021-09-29

**Authors:** Patrícia W. Ferreira, Paulo J. Nogueira, Miguel A. de Araújo Nobre, Carlos Moura Guedes, Francisco Salvado

**Affiliations:** 1 Unidade de Epidemiologia, Instituto de Medicina Preventiva e Saúde Pública, Faculdade de Medicina, Universidade de Lisboa, Avenida Professor Egas Moniz, Lisboa, Portugal; 2 Research, Development and Education Department, Maló Clinic, Avenida dos Combatentes, Lisboa, Portugal; 3 Clínica Universitária de Estomatologia, Faculdade de Medicina, Universidade de Lisboa, Avenida Professor Egas Moniz, Lisboa, Portugal; 4 Prosthodontics Department, Maló Clinic, Avenida dos Combatentes, Lisboa, Portugal; 5 Centro de Investigação Integrada Egas Moniz, Campus Universitário, Quinta da Granja, Caparica

**Keywords:** dental implants, complications, implant survival, bone loss

## Abstract

**Objective** This study aimed to investigate the impact of mechanical complications on outcome measures for implant dentistry.

**Materials and Methods** This case–control study included 282 patients with mechanical complications occurring in fixed prosthetic rehabilitation supported by immediate function implants with external connection (cases) and 282 individuals without mechanical complications (control). Pairing was performed for sex, age (range = 3 years), and follow-up months (range = 11 months). The primary outcome measure was implant survival, while the secondary outcome measures were marginal bone loss and biological complication parameters (peri-implant pathology, soft tissue inflammation, fistula formation, and abscess formation).

**Statistical Analysis** Cumulative implant survival was estimated by using life tables. Descriptive statistics with 95% confidence intervals (CI) and inferential statistics (Chi-square test) were performed to evaluate differences between cases and controls. The significance level was set at 5%.

**Results** The average follow-up duration was 8.5 years. Mechanical complications included prosthetic fracture ( *n* = 159), abutment loosening ( *n* = 89), prosthetic screw loosening ( *n* = 20), milled abutment ( *n* = 12), milled prosthetic screw ( *n* = 1), and decemented crown ( *n* = 1). Implant failure occurred in one patient from the control group, with survival rates of 100 and 99.6% for cases and controls, respectively ( *p* = 0.317). The average marginal bone loss was 1.72 (95% confidence interval [CI]: 1.60–1.84) for cases and 1.55 (95% CI: 1.45–1.65) for controls ( *p* = 0.068). Biological complications were observed in 90 patients, with significant differences between cases ( *n* = 54) and controls ( *n* = 36; *p* = 0.038).

**Conclusion** Mechanical complications did not significantly influence survival or marginal bone loss; nevertheless, there is a need for studies with longer follow-up duration. Mechanical complications also significantly influence the incidence of biological complications.

## Introduction

Edentulism is a debilitating condition that affects not only oral health, but also the impact on general health and quality of life. 1 The treatment of missing teeth is very important to overcome functional, esthetic, and social challenges and to improve the quality of life. 2

The first scientifically documented concept for dental implant treatment, the Brånemark System, included a two-stage surgical technique with intermediate healing periods. 3 Because of the discomfort, inconvenience, and anxiety that waiting periods impose to both patients and clinicians, 4 the immediate function rehabilitation technique has been widely used in implant dentistry. This technique involves placing the implant(s), abutment(s), and crown(s) in the same surgical procedure, which means that the implant immediately fulfills the requirements of masticatory function and esthetics, 5 with demonstrated high implant survival rates under well-defined circumstances. 6 7

Mechanical risk factors are determinants of complications or failure of prefabricated components caused by mechanical forces, while technical factors are complications or failures of a structure fabricated in the laboratory or its materials. Both risks play an important role in implant dentistry, as a source of time and financial resources loss. 8 Considering that the periodontal ligament is absent from the peri-implant structure, excess occlusal, functional, or parafunctional forces may lead to mechanical complications and adverse effects on the implants’ structural integrity. 9 This occlusal overloading can also lead to marginal bone loss due to the inability of the host tissues to accommodate excessive forces. 10

Biological and mechanical complications in implant-supported fixed dental prostheses are frequent over a 5-year observation period (33.6%), with fractures of the veneering material (13.5%), peri-implant pathology or soft tissue complications (8.5%), loss of access hole restoration (5.4%), abutment or screw loosening (5.3%), and loss of retention of cemented fixed dental prostheses (4.7%) as the most recurrent. 11 12 Implant failure is secondary to mechanical complications, including implant fracture, abutment screw fracture, and abutment fracture, 13 with implant fracture as the most serious and rare complication with a prevalence of 1.6%. 14 It implies implant removal and replacement by a larger diameter implant, with consequent delay of the prosthetic rehabilitation due to the necessity of overcoming a new osseointegration period. 12

Based on the existing literature on implant-supported restorations, there is a relative gap in the understanding of the effect of mechanical complications on long-term outcomes. Mechanical complications could potentially be a primary factor for implant failure through a direct effect due to implant fracture or marginal bone resorption, or through an indirect effect due to biological complications occurring secondary to mechanical complications. It is important to understand the effect of mechanical complications on implant survival, marginal bone resorption, and incidence of biological complications. The aim of this study was to fill this gap by analyzing the effect of mechanical complications on the outcome of implant-supported restorations.

## Materials and Methods

This case–control study was performed from 2016 to 2019 at a private rehabilitation center, (Maló Clinic, Lisbon, Portugal). The study was approved by a local ethics committee (Ethical Committee for Health, authorization no. 011-2012). Informed consent was obtained from all participants. The study population consisted of patients rehabilitated with dental implants (age range = 18–80 years) and selected from a database with registered mechanical complications. Some conditions that could compromise treatment success such as smoking and systemic conditions were considered and not excluded. There were 282 patients identified with mechanical complications occurring in fixed prosthetic rehabilitation supported by immediate function implants (cases). The date of implant surgery ranged from September 1997 to December 2006.

The sample size ( *n* = 282 in each group) enabled the detection of factors associated with survival, with an odds ratio (ψ) of 1.545 or greater in exposed relative to unexposed (controls) groups according to calculations (PS power and sample size calculation software, version 2.1.30, February 2003). 15 The remaining assumptions used in the calculation were the following:

- Probability of treatment failure among controls of 30% 16 17 18 19
- Correlation coefficient for exposure between matched exposed and unexposed subjects of 20% 16 17 18 19
- Ratio of matching controls per exposed subjects of 1:1
- Statistical significance level of 95% (type I error or α = 0.05)
- Statistical power of 80% (type II error or β = 20%)

A total of 282 patients with mechanical complications (cases) were matched for sex, age (within a range of 3 years), and follow-up time (within a range of 11 months) with the patients without mechanical complications (controls).

### Surgical Protocol

The patients’ medical history was examined, and clinical observations complemented with orthopantomography and computerized tomography scans were performed. Teeth were extracted when needed at the time of surgery before implant placement. A mucoperiosteal flap was raised at the ridge crest, relieving incisions on the buccal aspect of the molar area. The insertion of implants (external connection, Mk II, Mk III, Mk IV, NobelSpeedy; Nobel Biocare AB) followed standard procedures, except that underpreparation was used to achieve an insertion torque of at least 35 N∙cm before the final seating of implants. The preparation was typically performed by using a full drill depth with a 2-mm twist drill, followed by twist/step drills according to the manufacturer’s protocol. 20 21 The implant neck (Mk II, Mk III, or Mk IV implant) or the implant head (NobelSpeedy implant) was positioned at the bone level, and bicortical anchorage was established whenever possible. The implant diameter ranged from 3.3 to 4 mm, and the implant length ranged from 10 to 18 mm. The abutments were used according to the type of restoration (Cera-one, STR, multiunit straight and angulated abutments, Nobel Biocare AB). After closing and suturing the flap with 3–0 nonabsorbable sutures, the abutments were assessed by using a punch if needed, and impression copings were placed.

### Immediate Provisional Prosthetic Protocol

High-density acrylic resin (Heraeus Kulzer GmbH, Hanau, Germany) and titanium cylinder (Nobel Biocare AB) prostheses (complete or partial edentulous) or crowns (single tooth) were manufactured at the dental laboratory and inserted on the same day of surgery ( *n* = 465) to achieve immediate function.

### Final Prosthetic Protocol

Considering the patient’s preference, a metal-ceramic implant-supported fixed prosthesis with a titanium framework and all-ceramic crowns (Procera titanium framework, Procera crowns, Nobel Rondo ceramics, Nobel Biocare AB), or a metal-acrylic resin implant-supported fixed prosthesis with a titanium framework (Procera titanium framework; Nobel Biocare AB) and acrylic resin prosthetic teeth (Heraeus Kulzer GmbH) was used to replace the provisional prosthesis for the complete edentulous. For partial and single tooth rehabilitation, ceramic crown/prosthesis (Procera crowns, Nobel Rondo ceramics, Nobel Biocare AB) was inserted. In the final prosthesis, occlusion mimicked natural dentition.

### Outcome Measures

Outcome measures were evaluated yearly between implant insertion and the last clinical appointment for follow-up. The primary outcome measure was implant survival, which was evaluated based on the function of the implant as part of a prosthetic rehabilitation unit. The secondary outcome measures were marginal bone loss, which was measured at 5 years of follow-up, and complications. Considering marginal bone loss, periapical radiographs were obtained by using the parallel technique with a film holder (Super-Bite, Hawe-Neos, Switzerland) and an aiming device. Each periapical radiograph was scanned at 300 DPI by using a scanner (HP Scanjet 4890, HP Portugal, Paço de Arcos, Portugal), and the marginal bone level was assessed by using an image analysis software (Image J version 1.40g for Windows, National Institutes of Health, United States). The reference point for reading was the implant platform, which is the horizontal interface between the implant and the abutment. Marginal bone loss was defined as the difference in marginal bone level relative to the bone level at the time of surgery. Radiographs were accepted or rejected for evaluation based on the clarity of implant threads; a clear thread guaranteed both sharpness and an orthogonal direction of the radiographic beam toward the implant axis.

The following biological complication parameters were assessed: peri-implant pathology (probing pocket depth >4 mm, with concurrent presence of marginal bone loss and bleeding on probing/suppuration), soft tissue inflammation, fistula formation, and abscess formation. This evaluation was performed after surgical healing, every 6 months, and over at least 5 years of follow-up.

## Statistical Analysis

Descriptive statistics were performed on the variables of interest, and frequencies of mechanical complications, including prosthesis fracture, prosthetic decementation, abutment screw milling or loosening, and prosthetic screw milling or loosening, and biological complications, including inflammation, infection, and peri-implant pathology, were estimated. Implant survival was recorded as survival or failure and was estimated through life tables, mechanical, and biological complications were recorded as present or absent, and marginal bone loss was recorded through the average and 95% confidence interval (CI) using the patient as a unit of analysis. Inferentially, smoking status, systemic compromising status, implant survival, and the incidence of biological complications were compared between groups using the Chi-square test, with complementary analysis (Chi-square test and odds ratio) to assess the difference in the distribution of biological complications according to the timing of occurrence (less than 6 months or between 6 months and 1 year after the incidence of mechanical complications), and marginal bone loss at 5 years was compared between groups using the Mann–Whitney U test. Statistical analyses were performed by using the SPSS software (version 17.0; IBM, New York, United States). The significance level was set at 5%.

## Results

A total of 564 patients (330 women and 234 men) were included, with an average age of 52.6 years (range = 18–80 years) and with 175 patients who were smokers and 151 patients with a systemic condition. There were 167 single-tooth rehabilitations, 106 partial rehabilitations, and 291 complete edentulous rehabilitations. Regarding the opposing dentitions, 16 patients had a removable prosthesis, 144 patients had natural teeth, 108 patients had fixed prosthetics over natural teeth, 250 patients had a combination of natural teeth and implant-supported fixed prostheses, and 46 patients had a removable prosthesis. Mechanical complications included prosthetic fracture ( *n* = 159), abutment loosening ( *n* = 89), prosthetic screw loosening ( *n* = 20), milled abutment ( *n* = 12), milled prosthetic screw ( *n* = 1), and decemented crown ( *n* = 1). The distribution of mechanical complications in these cases is presented in Table 1 .

**Table 1:** Distribution of mechanical complications in the cases

Type of Mechanical Problems	Number of occurrences per patient	Number of occurrences per restoration	Number of recurring complications	Number of recurrences per restoration
Veneer fracture	159	Full-arch: 110 Partial: 12 Single 37	264	Full-arch: 240 Partial: 4 Single: 20
Prosthetic screw loosening	20	Full-arch: 13 Partial: 5 Single 2	33	Full-arch: 33
Milled prosthetic screw	1	Partial: 1	0	
Abutment screw loosening	89	Full-arch: 64 Partial: 6 Single 19	78	Full-arch: 62 Partial: 1 Single: 15
Milled abutment screw	12	Full-arch: 11 Partial: 1	12	Full-arch: 11 Partial: 1
Decemented crown	1	Partial: 1	3	Partial: 3
Total	282		390	
Note: Total number of nine patients with more than one incidence: *n* = 4 patients with fracture prosthesis and abutment screw loosening; *n* = 3 patients with fractured prosthesis and abutment screw milling; *n* = 1 patient with fractured prosthesis and prosthetic screw milling; *n* = 1 patient with fractured prosthesis and prosthetic screw loosening.				

The average follow-up period of the sample was 8.5 years. The distribution of patients with smoking habits was 88 (50.3%) and 87 (49.7%) individuals for cases and controls, respectively ( *p* = 0.500, Chi-square test); for systemic conditions, there were 71 (51.1%) and 68 (48.9%) patients for cases and controls, respectively ( *p* = 0.423, Chi-square test); and for history of periodontal disease, there were 161 (52.3%) and 147 (47.7%) patients for cases and controls, respectively ( *p* = 0.430, Chi-square test). No significant differences between cases and controls were observed in the distribution of smoking habits, systemic condition, or history of periodontitis.

Implant failure occurred in one patient from the control group after 70 months of follow-up, with survival rates of 100 and 99.6% for cases and controls, respectively ( Table 2 ). The difference in survival outcomes between the groups was not significant ( *p* = 0.317, Chi-square test).

**Table 2:** Life tables evaluating the cumulative survival rate (global sample, patients exposed to mechanical complications, and patients unexposed to mechanical complications)

Global sample						
Time	**Total number of patients**	**Failures**	**Lost to follow-up**	**Follow-up not completed**	**Survival rate (%)**	**Cumulative survival rate (%)**
**Placement–1 y**	564	0	0	0	100	100.0
**1–2** y	564	0	0	0	100	100.0
**2–3** y	564	0	0	0	100	100.0
**3–4** y	564	0	0	0	100.0	100.0
**4–5** y	564	0	0	0	100.0	100.0
**5–6** y	564	1	17	55	99.8	99.8
**6–7** y	491	0	11	146	100.0	99.8
**7–8** y	334	0	10	124	100.0	99.8
**8–9** y	200	0	7	113	100.0	99.8
**Patients with mechanical complications (cases)**						
**Time**	**Total number of patients**	**Failures**	**Lost to follow-up**	**Follow-up not completed**	**Survival rate (%)**	**Cumulative survival rate (%)**
**Placement–1** y	282	0	0	0	100	100.0
**1–2** y	282	0	0	0	100	100.0
**2–3** y	282	0	0	0	100	100.0
**3–4** y	282	0	0	0	100.0	100.0
**4–5** y	282	0	0	0	100.0	100.0
**5–6** y	282	0	7	29	100.0	100.0
**6–7** y	246	0	1	79	100.0	100.0
**7–8** y	166	0	2	66	100.0	100.0
**8–9** y	98	0	0	59	100.0	100.0
**Patients without mechanical complications (controls)**						
**Time**	**Total number of patients**	**Failures**	**Lost to follow-up**	**Follow-up not completed**	**Survival rate (%)**	**Cumulative survival rate (%)**
**Placement–1 y**	282	0	0	0	100	100.0
**1–2** y	282	0	0	0	100	100.0
**2–3** y	282	0	0	0	100	100.0
**3–4** y	282	0	0	0	100.0	100.0
**4–5** y	282	0	0	0	100.0	100.0
**5–6** y	282	1	10	26	99.6	99.6
**6–7** y	245	0	10	67	100.0	99.6
**7** y	168	0	8	58	100.0	99.6
**8–9** y	102	0	7	54	100.0	99.6

**Fig. 1:**
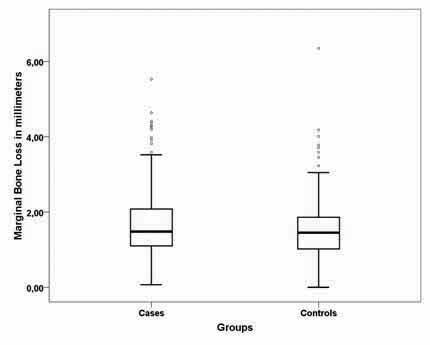
Boxplot of marginal bone loss at 5 years for cases (with mechanical complications) and controls (without mechanical complications). Box edges represent the first and third quartiles of data (25 and 75%, respectively, of all data collected); black line represents the median marginal bone loss registered for cases (1.48 mm) and controls (1.45 mm); whiskers represent all data not suspected to be outliers; dots represent data suspected of being outliers.

The average (95% CI) marginal bone loss registered for cases and controls was 1.72 (95% CI: 1.60–1.84) and 1.55 (95% CI: 1.45–1.65). The frequencies of marginal bone loss are shown in Table 3 and Fig. 1 . The difference in marginal bone loss between the cases and controls was not significant ( *p* = 0.068; Mann–Whitney U test).

**Table 3:** Marginal bone level for cases and controls at 5 years of follow-up

	Cases		Controls	
Mean (mm)	1.72		1.54	
Standard deviation (mm)	0.90		0.76	
Number	230		229	
Frequencies	*n*	%	*n*	%
0 mm	0	0.0	1	0.0
0.1–1.0 mm	47	20.4	62	27.1
1.1–2.0 mm	122	53.0	123	53.7
2.1–3.0 mm	38	16.5	34	14.8
>3.0 mm	23	10.0	9	3.9

Biological complications were observed in 90 patients (54 from the cases and 36 from the controls; Tables 4 and 5 ). The biological complications recorded were peri-implant pathologies ( *n* = 78 patients; 46 cases, 32 controls), fistula formation ( *n* = 2 patients; 1 case, 1 control), and abscess formation ( *n* = 10 patients; 7 cases, 3 controls), and the distribution of biological complications according to the type of mechanical complication and timing of occurrence (<6 months or >6 months after the incidence of a mechanical complication) are shown in Table 4 . A significant difference was observed in the incidence of biological complications between cases and controls ( *p* = 0.038, Chi-square test), with an odds ratio of 1.63 ( Table 5 ). Abscess and fistula formation were managed nonsurgically through prophylaxis (using chlorhexidine) and antibiotic therapy. Peri-implant pathology was managed nonsurgically ( *n* = 47 patients; 27 cases, 20 controls) through mechanical debridement and pocket irrigation with 0.2% chlorhexidine gel, or surgically ( *n* = 4 patients; 3 cases, 1 control) through an open flap surgical intervention to mechanically clean the implant surface, disinfecting the implant surface with 0.2% chlorhexidine, suturing, and medicating the patient with antibiotics. In 27 patients (16 cases, 11 controls), the interventions were unsuccessful through nonsurgical interventions ( *n* = 23 patients; 13 cases, 10 controls) or surgical interventions ( *n* = 4 patients; 3 cases, 1 control).

**Table 4:** Distribution of biological complications according to the timing of occurrence after the mechanical complication

	Biological complications <6 mo				Biological complications >6 mo			
**Mechanical complications**	**Abscess**	**Fistula**	**Peri-implant pathology**	**Total** **(per row)**	**Abscess**	**Fistula**	**Peri-implant pathology**	**Total** **(per row)**
Prosthetic screw loosening	1	0	1	2	0	0	1	1
Abutment screw loosening	1	1	5	7	0	0	15	15
Prosthesis fracture	3	0	7	10	0	0	19	19
Total (per column)	5	1	13	19	0	0	35	35

**Table 5:** Frequencies of biological complications in the exposed and unexposed groups

Mechanical complications		Biological complications		
		**Absent**	**Present**	**Total**
Absent (unexposed)	Number	246	36	282
	Within mechanical complications (%)	87.2	12.8	100.0
	Within biological complications (%)	52.0	40.0	50.1
	Total (%)	43.7	6.4	50.1
Present (exposed)	Number	227	54 ^a^	281
	Within mechanical complications (%)	80.8	19.2	100.0
	Within biological complications (%)	48.0	60.0	49.9
	Total (%)	40.3	9.6	49.9
Total	Number	473	90	563
	Total (%)	84.0	17.4	100.0
^a^ An odds ratio of 1.63 was estimated based on the figures presented.				

## Discussion

The results of this case–control study indicate that the exposure to mechanical complications in immediate loading protocols did not significantly impact implant survival after an average of 8.5 years of follow-up.

The effect of mechanical complications on successful outcomes of implant-supported rehabilitation is unclear. Salvi and Brägger, 8 in a systematic review of 35 publications with the purpose of understanding which mechanical/technical risk factors impacted implant-supported reconstructions, identified 10 mechanical/technical risk factors, including the history of mechanical/technical complications. The study concluded that none of the mechanical/technical risk factors for overloading had an impact on implant survival and success rates. 8 The present study supports this finding as mechanical/technical complications such as loosening or fracture of prosthetic components did not had a significant effect on survival, based on the nonsignificant difference in implant survival. On the other hand, previous studies have indicated an association between occlusal overloading, which is the primary cause of mechanical complications, 22 and possible late implant failure through marginal bone resorption. 10 The present study demonstrated that while there was no impact on implant survival, there was a potential deleterious influence on the long-term as observed when analyzing the clinical implications of both marginal bone resorption and incidence of biological complications.

The marginal bone loss after 5 years of follow-up revealed an increased bone loss in cases compared to controls; however, the difference was not statistically significant. Occlusal overloading, manifested through signs of mechanical complications, is the primary cause of biomechanical implant complications and may also disrupt the intricate bond between the implant surface and bone, leading to peri-implant bone loss and eventual implant failure. 10 According to Fu et al, 10 although the exact mechanism of peri-implant bone loss occurrence caused by occlusal overloading remains debatable due to confounding factors, it is obvious that a positive correlation between occlusal overloading and peri-implant marginal bone loss exists. Nevertheless, other researchers acknowledged that the effect of traumatic forces in peri-implant bone loss is poorly reported and provides limited evidence to support a cause-effect relationship considering the strength of a clinically relevant traumatic occlusal force. 23 In the present study, the marginal bone loss trend in cases (with 23 patients in cases compared to 9 patients in controls who had marginal bone loss of >3 mm at 5 years) might have a significant clinical impact. This marginal bone loss can be considered pathologic because “physiological” bone remodeling around implants was previously described to be approximately 1 mm during the first year of function and <0.2 mm per year subsequently. 24 Furthermore, the difference of 0.18 mm in marginal bone loss at 5 years of follow-up between the two groups clinically translates to roughly half an implant’s thread. Based on this difference, it may be hypothesized that the bone loss pattern in cases could have a significant impact on long-term follow-up considering functional and esthetic outcomes, with a potential visible abutment/crown transition that implies prosthetic rehabilitation failure. This hypothesis needs to be confirmed through studies with longer follow-up periods.

Given that prosthetic treatment takes place at the implant/abutment level, it became evident that the implant restoration process contributes significantly to the prognosis and peri-implant disease experience. 24 The present study reported a significant effect of mechanical complications on the incidence of biological complications (with a 63% increased odds ratio), a result that is supported by the current understanding of peri-implant disease etiopathogenesis. Epidemiologically, peri-implant pathology is considered a multifactorial disease with several nonsufficient and non-necessary causes, containing factors of biological and biomechanical origin that act independently or in association. 25 Nevertheless, other factors may contribute to the susceptibility or progression of peri-implant disease, such as patient’s health, smoking habits, the presence or preexistence of periodontal disease, the type of implant and its surface, and the quality of the existing bone. 24 26 Recent meta-analyses registered a higher incidence of peri-implant pathology in periodontitis-susceptible patients, 27 even under regular supportive postimplant treatment, 28 and smokers with significant effect on the incidence of postoperative infections, marginal bone loss, and implant failure rate. 29 30 31 Lin et al, 28 in a systematic review and meta-analysis of 13 studies to investigate if periodontal disease could still be a risk indicator for peri-implant health under supportive postsurgical treatment, estimated an increased marginal bone loss irrespective of the implant surface, and lower survival rates, increased pocket depth, and bleeding on probing in rough surface implants for patients with history of periodontitis. Chrcanovic et al, 31 in a systematic review and meta-analysis of 107 studies to estimate the effect of smoking on dental implants, reported a significant effect of smoking on implant failure (risk ratio of 2.23) and marginal bone loss (mean increase of 0.32 mm), which are two of the outcomes evaluated in the present study. Furthermore, certain systemic diseases, medications, radiotherapy, and behavioral factors, such as inefficient oral hygiene and lack of compliance with periodontal maintenance therapy, appear to significantly increase the risk of peri-implant pathology. 32 In the present study, all three conditions that could impact the outcome negatively were prevalent, with 54.6% of patients with history of periodontitis, 31% of patients who smoke, and 24.7% of patients with systemic conditions. However, because the distribution of patients with a history of periodontitis, smoking habits, and systemic condition between groups was not significantly different, it was suggested that mechanical complications may represent a risk indicator for the incidence of biological complications, rather than a confounder. A similar result was reported in a large case–control study to evaluate risk indicators for peri-implant disease, and the presence of mechanical complications such as prosthetic screw loosening, abutment screw loosening, or prosthetic passive misfit implied a 5.9-fold increase in the odds for peri-implant pathology 25 and consequent inclusion in a risk score to predict this disease. 33 34 Nevertheless, the fact that the results of the present study were not controlled for the presence of bacterial plaque implies both interpreting this result with caution and performing studies with stronger design to establish causality.

While there is no clear and long-term evidence of the impact of mechanical complications on implant treatment, clinicians must be aware of all patient-related conditions, providing the best treatment possible and preventing complications. An appropriate and individualized treatment plan, combined with regular routine appointments to identify warning signs, provides greater treatment success. Mechanical complications not only affect the clinical outcome, but also negatively impact the patients’ quality of life, translating into a source of frustration for both clinicians and patients, with the necessity of significant investment in terms of service, maintenance, costs, and time. 35

The limitations of the present study include the lack of randomization and its retrospective design. Another limitation is the fact that potential risk indicators or confounding factors, such as bacterial plaque and frequency of maintenance appointments, were not analyzed. Long-term longitudinal studies with multivariable analysis that include competing risk indicators and confounders should be performed to investigate both the effect and impact of the occurrence of mechanical complications on the outcome of implant-supported rehabilitations.

In conclusion, within the limitations of the present study, the occurrence of mechanical complications did not significantly impact implant survival or marginal bone loss at 5 years of follow-up, but did impact the incidence of biological complications, with a greater incidence in cases. Additional studies with longer follow-up periods are necessary to assess the implication of the incidence of biological complications and marginal bone loss pattern exhibited by patients with mechanical complications on the outcome of implant-supported rehabilitations.
